# Supplementary material for: Fabrication of Ultrafine PPS Fibers with High Strength and Tenacity via Melt Electrospinning
Source: Polymers (Basel). 2019 Mar 20;11(3):530. doi: 10.3390/polym11030530 (PMC6473442; doi:10.3390/polym11030530)
Supplement: Supplementary file 1 [file polymers-11-00530-s001.zip › polymers-453962/supporting information.docx]

Supporting Information

Fabrication of ultrafine PPS fibers with high strength and tenacity via melt electrospinning

*Zuo-Ze Fan, Hong-Wei He, Xu Yan, Ding Yuan, Fang-Gang Ning, Xin Ning*

*Industrial Research Institute of Nonwovens & Technical Textiles, College of Textiles &Clothing, Qingdao University, Qingdao 266071, China*

**
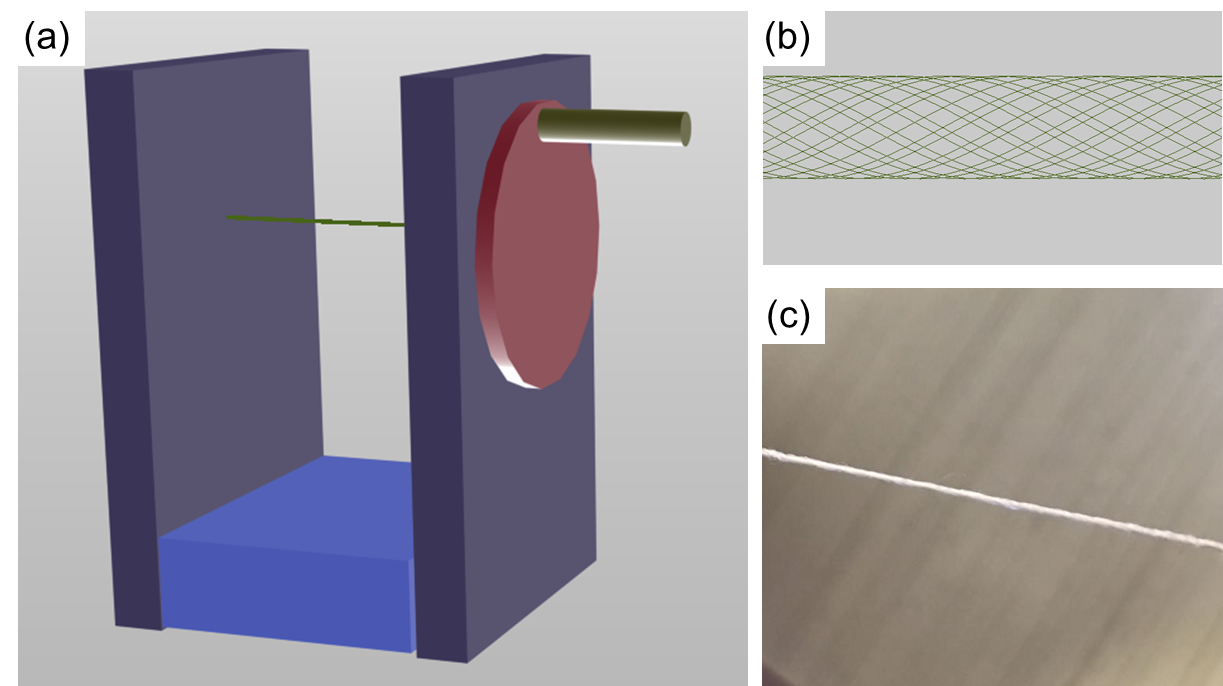
**

Figure.S1 (a) An illustration of home-made twisted device and (b) structural model of twisted rope. (c) An image of twisted rope.
